# Analysis of the Impact of Known *SPINK1* Missense Variants on Pre-mRNA Splicing and/or mRNA Stability in a Full-Length Gene Assay

**DOI:** 10.3390/genes8100263

**Published:** 2017-10-10

**Authors:** Hao Wu, Arnaud Boulling, David N. Cooper, Zhao-Shen Li, Zhuan Liao, Claude Férec, Jian-Min Chen

**Affiliations:** 1 Department of Gastroenterology, Changhai Hospital, The Second Military Medical University, Shanghai 200433, China; wuhao_lnly@163.com (H.W.); zhaoshenli@hotmail.com (Z.-S.L.); 2 Institut National de la Santé et de la Recherche Médicale (INSERM), U1078, Brest 29238, France; arnaud.boulling@hotmail.fr (A.B.); claude.ferec@univ-brest.fr (C.F.); 3 Etablissement Français du Sang (EFS)—Bretagne, Brest 29200, France; 4 Shanghai Institute of Pancreatic Diseases, Shanghai 200433, China; 5 Institute of Medical Genetics, School of Medicine, Cardiff University, Cardiff CF14 4XN, UK; cooperDN@cardiff.ac.uk; 6 Faculté de Médecine et des Sciences de la Santé, Université de Bretagne Occidentale (UBO), Brest 29238, France; 7 Laboratoire de Génétique Moléculaire et d’Histocompatibilité, Centre Hospitalier Universitaire (CHU) Brest, Hôpital Morvan, Brest 29200, France

**Keywords:** chronic pancreatitis, full-length gene assay, minigene assay, missense variant, mRNA stability, pre-mRNA splicing, *SPINK1* gene

## Abstract

It is increasingly appreciated that missense variants may not only alter protein structure and function but may also influence pre-mRNA splicing and/or mRNA stability. Here we explore this issue in the context of currently known *SPINK1* missense variants using a full-length gene assay. We demonstrated that 4 (17%) out of 24 variants tested significantly reduced pre-mRNA splicing and/or stability as compared with the wild-type. However, since the strongest effect observed was a 23% reduction from normal, the contribution of *SPINK1* missense variants to the clinical phenotype through an impact on mRNA processing alone may be relatively minor compared with their effects in relation to protein structure/function.

## 1. Introduction

Recently, it was reported that that up to 10% of known disease-associated missense variants alter pre-mRNA splicing [1]. In addition, missense variant-containing transcripts may also be less stable as compared to wild-type, if for example the variants facilitate the formation of alternative mRNA secondary structures [2]. *SPINK1* (encoding pancreatic secretory trypsin inhibitor; OMIM #167790) is one of the most studied genes predisposing to chronic pancreatitis [3]. We have recently provided both in vitro and in silico evidence [4] against a significant effect of the *SPINK1* c.194G>A variant on pre-mRNA splicing, contrary to previous claims by Beer and Sahin-Tóth [5]. The key difference between these studies was that the former used a full-length gene assay to assess pre-mRNA splicing, whereas the latter used a minigene assay; full-length gene assays are generally superior to minigene assays because pre-mRNA splicing is highly dependent upon the genomic context [6]. We were thus able to conclude that the pathogenic effect of the c.194G>A missense variant (p.Arg65Gln) was exerted exclusively via its impact on protein secretion, as previously reported [7,8], rather than through an influence on pre-mRNA splicing.

Prompted, however, by the aforementioned publication of Soemedi et al. [1], we sought to analyze the effect of all currently known *SPINK1* missense variants on pre-mRNA splicing and/or mRNA stability by means of our previously established full-length gene assay [4].

## 2. Materials and Methods

### 2.1. Mutagenesis

The 23 additional *SPINK1* missense variants as well as three synonymous variants logged in the Genetic Risk Factors in Chronic Pancreatitis database [9]; Table 1) were introduced separately into the pcDNA3.1/V5-His-TOPO vector harboring the wild-type full-length 7-kb *SPINK1* genomic sequence (including all four exons plus the three introns of the gene [10]) with Agilent’s QuikChange II XL Site-Directed Mutagenesis Kit (Les Ulis, France), essentially as previously described [11]. The sequences of the mutagenesis primers are available upon request. The successful introduction of the desired variants was confirmed by direct sequencing; the primers used for this purpose included 5′-TAATACGACTCACTATAGGG-3′ (for exon 1 variants), 5′-TGCCTGATTCATTTCCAGCAG-3′ (for exon 2 variants), 5′-TCAGAAGGGCCATAGGACTT-3′ (for exon 3 variants), and 5′-AGAGGCATCAGGAGCAAAAG-3′ (for exon 4 variants).

### 2.2. Cell Culture, Transfection, RNA Extraction, and Reverse Transcription

Human embryonic kidney 293T (HEK293T) cells were cultured in Dulbecco’s modified Eagle medium with 10% fetal calf serum. Twenty-four hours prior to transfection, 3.5 × 10^5^ cells were seeded per well of 6-well plates. For qualitative reverse transcription-PCR (RT-PCR) analyses (see Section 2.3), 1 µg wild-type or variant *SPINK1* expression vector, mixed with 3 mL Lipofectamine 2000 Reagent (Life Technologies, Courtaboeuf, France), was used for transfection per well. For quantitative RT-PCR analyses (see Section 2.4), 500 ng wild-type or variant *SPINK1* expression vector plus 500 ng pGL3-GP2 minigene [10] were mixed with 3 mL Lipofectamine 2000 Reagent for transfection. Forty-eight hours after transfection, total RNA was extracted using the RNeasy Mini Kit (Qiagen, Courtaboeuf, France). Reverse transcription was performed with 1 μg total RNA and 20mer-oligo(dT) by means of the SuperScript III Reverse Transcriptase kit (Life Technologies) in accordance with the manufacturer’s instructions. The resulting complementary DNAs (cDNAs) were treated with 2U Ribonuclease H (Life Technologies) at 37 °C for 20 min to degrade the remaining RNA.

### 2.3. Qualitative RT-PCR Analyses and Sequencing of the Resulting Products

Qualitative RT-PCR was performed in a 25-μL reaction mixture containing 12.5 μL HotStarTaq Master Mix (Qiagen), 0.4 μM each primer (forward: 5′-GGAGACCCAAGCTGGCTAGT-3′, located within the 5′-untranslated region encoded by the pcDNA3.1/V5-His-TOPO vector; reverse: 5′-AGACCGAGGAGAGGGTTAGG-3′, located within the V5 epitope sequence encoded by the pcDNA3.1/V5-His-TOPO vector) and 1 μL cDNA. The PCR program had an initial denaturation at 95 °C for 15 min, followed by 30 cycles of denaturation at 94 °C for 45 s, annealing at 58 °C for 45 s, extension at 72 °C for 5 min, with a final extension step at 72 °C for 10 min. PCR products were cleaned by ExoSAP-IT (Life Technologies) and sequenced using the BigDye Terminator v1.1 Cycle Sequencing Kit (Life Technologies), the primer used for sequencing was the aforementioned 5′-GGAGACCCAAGCTGGCTAGT-3’.

### 2.4. Quantitative RT-PCR Analyses

These were performed essentially as previously described [10,12]. In brief, wild-type or variant *SPINK1* expression vector was co-transfected with the previously constructed pGL3-GP2 minigene [10]. The target (*SPINK1*) and reference (i.e., pGL3-GP2 minigene) genes were PCR-amplified separately; *SPINK1* expression was determined using pGL3-GP2 minigene expression as a reference by means of the ΔΔ*C*_t_ method in accordance with Pfaffl’s efficiency-corrected calculation model [13].

Quantitative RT-PCR was performed in a 25 µL mixture containing 12.5 µL HotStarTaq Master Mix Kit (Qiagen), 1 µL 1:25 diluted cDNA, 0.5 µM SYTO9 (Life Technologies), and 0.3 µM each primer; the primers for qualitative RT-PCR analyses of the *SPINK1* gene (see Section 2.3) were used for analyzing the *SPINK1* gene whilst the primers used for analyzing the reference gene were 5′-ACTGTTGGTAAAGCCACCAT-3′ (forward) and 5’-TGTATCTTATCATGTCTGCTCGAA-3’ (reverse). The PCR program had an initial denaturation step at 95 °C for 15 min, followed by 40 cycles of denaturation at 94 °C for 45 s, annealing at 55 °C for 30 s, and extension at 72 °C for 30 s. Quantitative analysis of each RT-PCR amplification was performed in triplicate on a Lightcycler 480II (Roche, Paris, France) using ‘relative quantification’ and ‘second derivative maxima’ options.

The difference between the expression levels of a *SPINK1* variant and the wild-type sequence (results from three to five independent transfection experiments) was assessed for significance by the Student *t*-test. The difference was regarded as being statistically significant when the *p*-value was ≤0.05.

## 3. Results and Discussion

**Figure 1:**
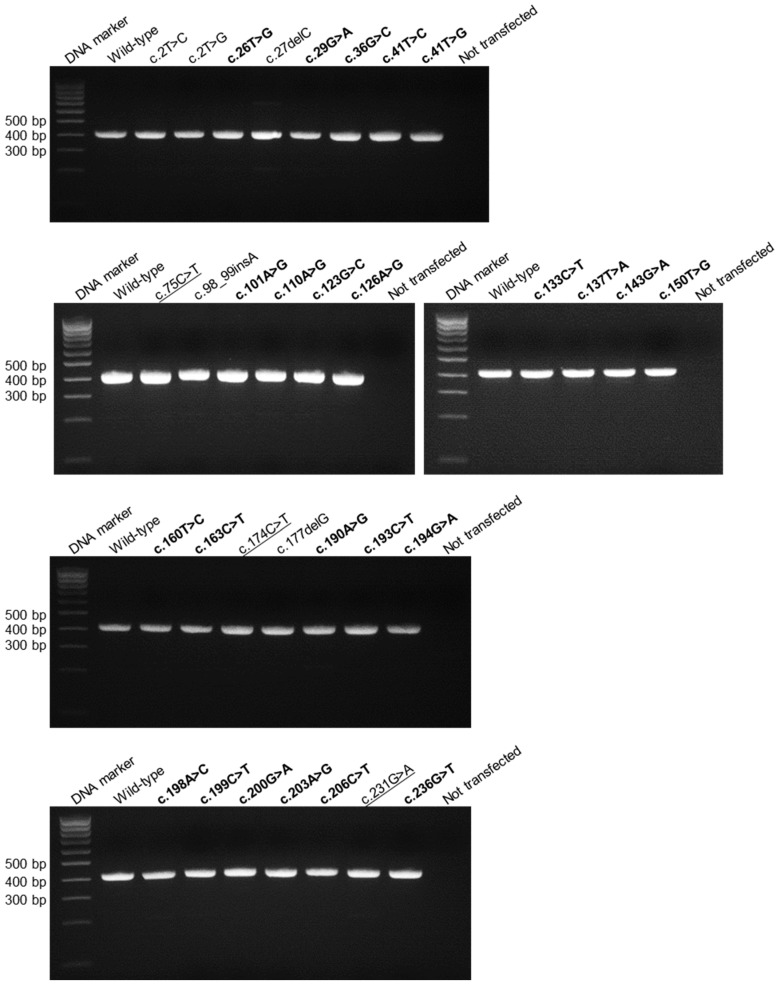
Qualitative RT-PCR analyses of HEK293T cells transfected with the full-length gene expression constructs carrying various *SPINK1* exonic variants. The missense and synonymous variants under study (Table 1) are highlighted in bold and underlining, respectively. The remaining variants, all of which are clearly loss-of-function variants (i.e., translational initiation codon or frameshifting variants), were not discussed in this study. Note that the previously studied c.194G>A variant [4] was also included for the reason of completeness.

Qualitative RT-PCR analyses of mRNAs from HEK293T cells transfected with the 23 missense variants and 3 synonymous variants invariably yielded a single band of similar size to that of the wild-type (Figure 1). Sequencing of the 26 corresponding RT-PCR products revealed that all were correctly spliced as per the wild-type. When quantitative RT-PCR analyses were performed, a statistically significant lower mRNA expression level was observed with 4 of the 23 missense variants but not with any of the synonymous variants (Table 1). In this regard, it is pertinent to point out that the reliability of our quantitative RT-PCR method had been experimentally validated in our previous study [4].

The decrease in mRNA expression noted for the four missense variants may be explicable in terms of two non-mutually exclusive mechanisms. Firstly, these variants could have led to the production of aberrantly spliced transcripts but these aberrant transcripts may have been subjected to rapid degradation by cellular defense mechanisms [14], rendering them undetectable by qualitative RT-PCR analysis. Secondly, and as mentioned above, correctly spliced variant-containing transcripts may be less stable as compared to wild-type through for example the formation of alternative mRNA secondary structures [2]. Whichever, the two different mechanisms are indistinguishable by our quantitative RT-PCR analysis.

Irrespective of the underlying biological mechanisms responsible, our systematic mRNA expression analysis of known *SPINK1* missense variants is suggestive of the existence of a new layer of functional deficiency, at least in the case of two specific missense variants (i.e., c.190A>G (p.Asn64Asp) and c.199C>T (p.Arg67Cys)), the pathogenicity of which had already been established (Table 1). (N.B. The pathogenicity classifications of all 24 *SPINK1* missense mutations (Table 1) were based essentially upon the analysis of in vitro-expressed mutant proteins in terms of their secretion and/or enzymatic activity.) Thus, the pathogenicity of these two variants may be seen to emanate from a combination of defective mRNA expression and protein function. By contrast, the other two missense variants characterized by a significant influence on pre-mRNA splicing and/or mRNA stability were previously classified as being either non-pathogenic or ‘protective’ (Table 1). Our new data may therefore prompt a re-consideration of these initial variant classifications. Specifically, the ‘non-pathogenic’ c.29G>A (p.Ser10Asn) variant may actually serve to increase the risk of chronic pancreatitis through its impact on mRNA expression and/or stability. As for the c.203A>G (p.Gln68Arg) variant, any ‘protective’ effect due to the significantly increased secretion of the mutant protein may be partly compensated for by its negative effect on pre-mRNA splicing and/or stability. This notwithstanding, two caveats should be borne in mind. First, the strongest observed effect on pre-mRNA splicing and/or stability was a 23% reduction from normal (c.199C>T). Whether such decreases are meaningful in a pathological context, particularly when we consider that the alleles responsible occur only in the heterozygous state in their respective carriers, is unclear. Second, the analyses should ideally be performed in a human pancreatic acinar cell line, which unfortunately is not yet available.

## 4. Conclusions

In summary, we demonstrate that four (~17%) of the currently known 24 *SPINK1* missense variants exert a significant impact on pre-mRNA splicing and/or mRNA stability. However, since the strongest effect observed was a 23% reduction from normal, the contribution of *SPINK1* missense variants to the clinical phenotype through their impact on mRNA processing is likely to be relatively minor. Finally, to the best of our knowledge, this study is the first to comprehensively analyze the consequences of all known missense mutations in a given gene on mRNA expression/stability in the context of a full-length gene assay. It remains to be seen if comparable results will be obtained for missense variants associated with other diseases.

## Figures and Tables

**Table 1:** Quantitative reverse-transcription PCR (RT-PCR) analyses of mRNA expression from HEK293T cells transfected with *SPINK1* missense variants.

Exon	Variant		Relative mRNA Expression ^a^		Pathogenicity Classification ^b^
	Nucleotide Change	Amino Acid Change	Mean ± SD	*p*-Value	
*Missense*					
1	c.26T>G	p.Leu9Arg	94.3 ± 8.0	0.248	Unknown
1	c.29G>A	p.Ser10Asn	80.0 ± 4.4	0.003	Non-pathogenic
1	c.36G>C	p.Leu12Phe	96.4 ± 9.8	0.517	Non-pathogenic
1	c.41T>C	p.Leu14Pro	91.5 ± 5.9	0.064	Pathogenic
1	c.41T>G	p.Leu14Arg	103.6 ± 12.9	0.621	Pathogenic
3	c.101A>G	p.Asn34Ser	103.8 ± 7.6	0.482	Non-pathogenic
3	c.110A>G	p.Asn37Ser	99.0 ± 6.2	0.800	Non-pathogenic
3	c.123G>C	p.Lys41Asn	97.0 ± 4.0	0.315	Pathogenic
3	c.126A>G	p.Ile42Met	108.2 ± 9.4	0.270	Unknown
3	c.133C>T	p.Pro45Ser	94.7 ± 6.0	0.266	Unknown
3	c.137T>A	p.Val46Asp	100.1 ± 2.7	0.954	Unknown
3	c.143G>A	p.Gly48Glu	106.0 ± 11.6	0.379	Pathogenic
3	c.150T>G	p.Asp50Glu	98.8 ± 5.0	0.700	Pathogenic
3	c.160T>C	p.Tyr54His	100.5 ± 15.7	0.953	Pathogenic
3	c.163C>T	p.Pro55Ser	105.9 ± 8.4	0.355	Non-pathogenic
3	c.190A>G	p.Asn64Asp	82.2 ± 4.1	0.017	Pathogenic
3	c.193C>T	p.Arg65Trp	97.8 ± 4.7	0.505	Unknown
3	c.194G>A ^c^	p.Arg65Gln	108.5 ± 10.4	0.295	Pathogenic
4	c.198A>C	p.Lys66Asn	97.5 ± 4.4	0.425	Pathogenic
4	c.199C>T	p.Arg67Cys	77.1 ± 10.1	0.007	Pathogenic
4	c.200G>A	p.Arg67His	94.3 ± 9.7	0.323	Pathogenic
4	c.203A>G	p.Gln68Arg	87.4 ± 7.0	0.037	Protective
4	c.206C>T	p.Thr69Ile	106.8 ± 7.1	0.240	Pathogenic
4	c.236G>T	p.Cys79Phe	110.0 ± 4.5	0.062	Pathogenic
*Synonymous*					
2	c.75C>T	p.Ser25=	90.2 ± 8.1	0.093	Unknown
3	c.174C>T	p.Cys58=	96.6 ± 4.2	0.291	Unknown
4	c.231G>A	p.Gly77=	93.3 ± 8.3	0.297	Unknown

^a^ Relative to the wild-type value, which was set to 100 (results from three to five independent transfection experiments). SD, standard deviation. Variants whose expression levels were statistically different from wild-type (i.e., *p* < 0.05) are highlighted in blue. ^b^ In accordance with the Genetic Risk Factors in Chronic Pancreatitis database [9]. ^c^ Previously analyzed by means of the full-length gene assay [4].
